# SARS-CoV-2 infection alkalinizes the ERGIC and lysosomes through the viroporin activity of the viral envelope protein

**DOI:** 10.1242/jcs.260685

**Published:** 2023-03-24

**Authors:** Wen-An Wang, Amado Carreras-Sureda, Nicolas Demaurex

**Affiliations:** Department of Cell Physiology and Metabolism, University of Geneva, Geneva 1211, Switzerland

**Keywords:** COVID-19, Coronavirus, Ion channel, Viroporin, Virus–host interactions

## Abstract

The coronavirus SARS-CoV-2, the agent of the deadly COVID-19 pandemic, is an enveloped virus propagating within the endocytic and secretory organelles of host mammalian cells. Enveloped viruses modify the ionic homeostasis of organelles to render their intra-luminal milieu permissive for viral entry, replication and egress. Here, we show that infection of Vero E6 cells with the delta variant of the SARS-CoV-2 alkalinizes the endoplasmic reticulum (ER)–Golgi intermediate compartment (ERGIC) as well as lysosomes, mimicking the effect of inhibitors of vacuolar proton ATPases. We further show the envelope protein of SARS-CoV-2 accumulates in the ERGIC when expressed in mammalian cells and selectively dissipates the ERGIC pH. This viroporin action is prevented by mutations of Val25 but not Asn15 within the channel pore of the envelope (E) protein. We conclude that the envelope protein acts as a proton channel in the ERGIC to mitigate the acidity of this intermediate compartment. The altered pH homeostasis of the ERGIC likely contributes to the virus fitness and pathogenicity, making the E channel an attractive drug target for the treatment of COVID-19.

## Teaser

**Summary:** SARS-CoV2 infection neutralizes the pH of the ERGIC due to the proton channel activity of the viral envelope protein, which is prevented by mutations at Val25.

## INTRODUCTION

The severe acute respiratory syndrome-coronavirus 2 (SARS-CoV-2), the agent of the dramatic ongoing COVID-19 pandemic, is the third coronavirus to cause severe respiratory disease in humans. SARS-CoV-2 propagates more efficiently than the highly pathogenic human coronaviruses SARS-CoV and MERS-CoV that emerged in 2002 and 2012, respectively (Goyal et al., 2022). Up to 20% of hospitalized infected post-COVID patients require respiratory support for acute respiratory distress syndrome (ARDS) triggered by a cytokine storm. Lung damage, intravascular coagulation and severe T cell lymphopenia characterize late-stage SARS-CoV-2 infection, with viral toxicity and hyper-inflammation both contributing to pathogenicity. A significant proportion of infected patients suffer from multisystemic effects cumulating into long-COVID symptoms that can be highly debilitating (Silva Andrade et al., 2021). Current therapeutic strategies combine antiviral drugs and immune modulators (Harrison, 2020), but are hampered by a lack of information on the molecular and cellular mechanisms of SARS-CoV-2 infection, putting intense pressure on prevention measures that rely on the worldwide distribution and administration of safe and effective vaccines.

The SARS-CoV-2 genome encodes for four structural proteins required to produce a complete infectious viral particle and 16 non-structural proteins that drive viral replication and contribute to viral pathogenicity. The spike (S) structural protein mediates virus entry via attachment to the angiotensin-converting enzyme (ACE2) receptor after priming by cellular proteases (Hoffmann et al., 2020). The nucleocapsid (N) protein binds the single-stranded, positive-sense viral RNA genome and organizes its replication. The membrane (M) and envelope (E) proteins drive virus assembly and budding. Once bound to cell surface ACE2 receptors, the S protein requires proteolytic cleavage by host cell proteases to drive the fusion of viral and cellular membranes. Depending on protease availability and cell type, the S protein can be cleaved at the plasma membrane (PM) by the serine protease TMPRSS2 (Hoffmann et al., 2020) or in endosomes by the cysteine proteases cathepsins B and L (Mingo et al., 2015; Smieszek et al., 2020). Direct PM entry is more efficient and is the preferred route in lung cells expressing TMPRSS2 (Hoffmann et al., 2020). Endocytosis is the default entry route and requires an acidic pH in the lumen of endosomes to activate cathepsin L (Simmons et al., 2005). Membrane fusion releases the viral genome into the host cell cytoplasm, generating viral proteins required for RNA synthesis and for the formation of intracellular double-membrane structures derived from the endoplasmic reticulum (ER) that serve as a scaffold for viral replication and as protection from antiviral host cell responses (Knoops et al., 2008).

Upon translation, the S, E and M structural proteins insert into the membrane of the ER and drive viral assembly in the ER–Golgi intermediate compartment (ERGIC). The virions then bud into the ERGIC lumen and accumulate in large vesicles to reach the PM and egress (Ruch and Machamer, 2012; Ulasli et al., 2010). Assembled β-coronaviruses have also been shown to egress through lysosomal exocytosis (Ghosh et al., 2020). SARS-COV-2 E and M redirect S to the ERGIC and are required for the optimal production of viral-like particles (Boson et al., 2021). The E and M proteins interact to drive membrane bending and scission (Ruch and Machamer, 2012), and exogenous expression of E generates tubular convoluted membranes mimicking those of infected cells (Raamsman et al., 2000). Combined E and M expression is sufficient to generate viral-like particles (Baudoux et al., 1998), and deleting the E gene reduces SARS-CoV infectivity, leading to intracellular accumulation of virions with aberrant material (DeDiego et al., 2007). Most of the E protein, however, does not incorporate in the virus, suggesting that it sustains infection by altering host cell functions.

The SARS-CoV-2 E protein (UniProtKB P59637) is a small 75-amino-acid protein, containing a predicted amphipathic transmembrane α-helix followed by a cluster of positively charged residues, two signature motifs that are characteristic of viral ion channels, also known as viroporins (Hyser and Estes, 2015). The E protein is the most conserved of SARS-CoV structural proteins, with 100% identity between SARS-CoV-2 and a CoV isolated from a Malayan pangolin, the likely intermediate host in the COVID-19 pandemic (Xiao et al., 2020). All CoV E proteins studied so far have ion channel activity and are inhibited by micromolar concentrations of hexamethylene amiloride (HMA) (Surya et al., 2015; Verdià-Bàguena et al., 2012; Wilson et al., 2006). Nuclear magnetic resonance (NMR) spectroscopy of SARS-CoV E revealed a pentameric channel (Pervushin et al., 2009; Surya et al., 2018) (PDB ID: 5X29) with residues Asn15 and Val25 contributing to ion conductance and oligomerization (Pervushin et al., 2009). The E protein of avian infectious bronchitis increases the pH of the Golgi (Westerbeck and Machamer, 2019), and the E protein of SARS-CoV functions as a Ca^2+^-permeable channel in artificial membranes (Nieto-Torres et al., 2015). The Ca^2+^ channel activity of SARS-CoV promotes viral replication and pathogenesis by disrupting the host Ca^2+^ signalling pathways (Hyser and Estes, 2015), and loss of channel function reduced virus fitness and pathogenicity (Regla-Nava et al., 2015). The structure of the transmembrane domain of the SARS-COV-2 E protein has been determined by solid-state NMR spectroscopy at 2.1-Å resolution (Mandala et al., 2020). The transmembrane domain reconstituted into ERGIC-mimetic lipid bilayers forms a five-helix bundle surrounding a narrow and partially dehydrated pore, consistent with its predicted channel function. In this pentamer model, the dehydrated pore is lined by valine and leucine residues and constricted by Asn15 (Mandala et al., 2020), consistent with electrophysiological data showing that mutation of Asn15 and Val25 abolish the cation conductance of SARS-CoV E (Verdià-Bàguena et al., 2012).

The viroporin function of the E protein of SARS-CoV-2 was recently established by electrophysiological recordings. Membrane currents carried by Na^+^ and K^+^ were recorded in planar lipid bilayers containing recombinant E (Xia et al., 2021) and in cells ectopically expressing E lacking its ER retention sequence and bearing a Golgi export sequence, to ensure its expression at the plasma membrane (Cabrera-Garcia et al., 2021). These results indicate that the E protein of SARS-CoV-2 forms an ion channel that is permeable to monovalent cations. The currents were sensitive to changes in extracellular pH (corresponding to changes in the pH of the ERGIC lumen) and expression of a tagged SARS-CoV-2 E increased the pH reported by an acidophilic dye (Cabrera-Garcia et al., 2021), indicating that E dissipates the pH of acidic organelles, as expected from its viroporin activity.

Here, we study the impact of SARS-CoV2 infection on the pH homeostasis of intracellular compartments of its host mammalian Vero E6 cells. By recording the pH within the cytoplasm, ERGIC and lysosome with genetically encoded pH probes after SARS-CoV2 infection or following ectopic expression of the SARS-CoV-2 E protein, we show that viral infection deacidifies both the ERGIC and lysosomes, whereas expression of the E protein alone increases the ERGIC pH, an effect that was not observed with an E protein bearing the V25F or N15A/V25F E mutations but that persisted in the single N15A mutant. The E protein therefore acts as a viroporin to alkalinize the ERGIC during viral infection, and likely contributes to virus pathogenicity.

## RESULTS

### Validation of a genetically encoded pH indicator targeted to the ERGIC

**Fig. 1.**
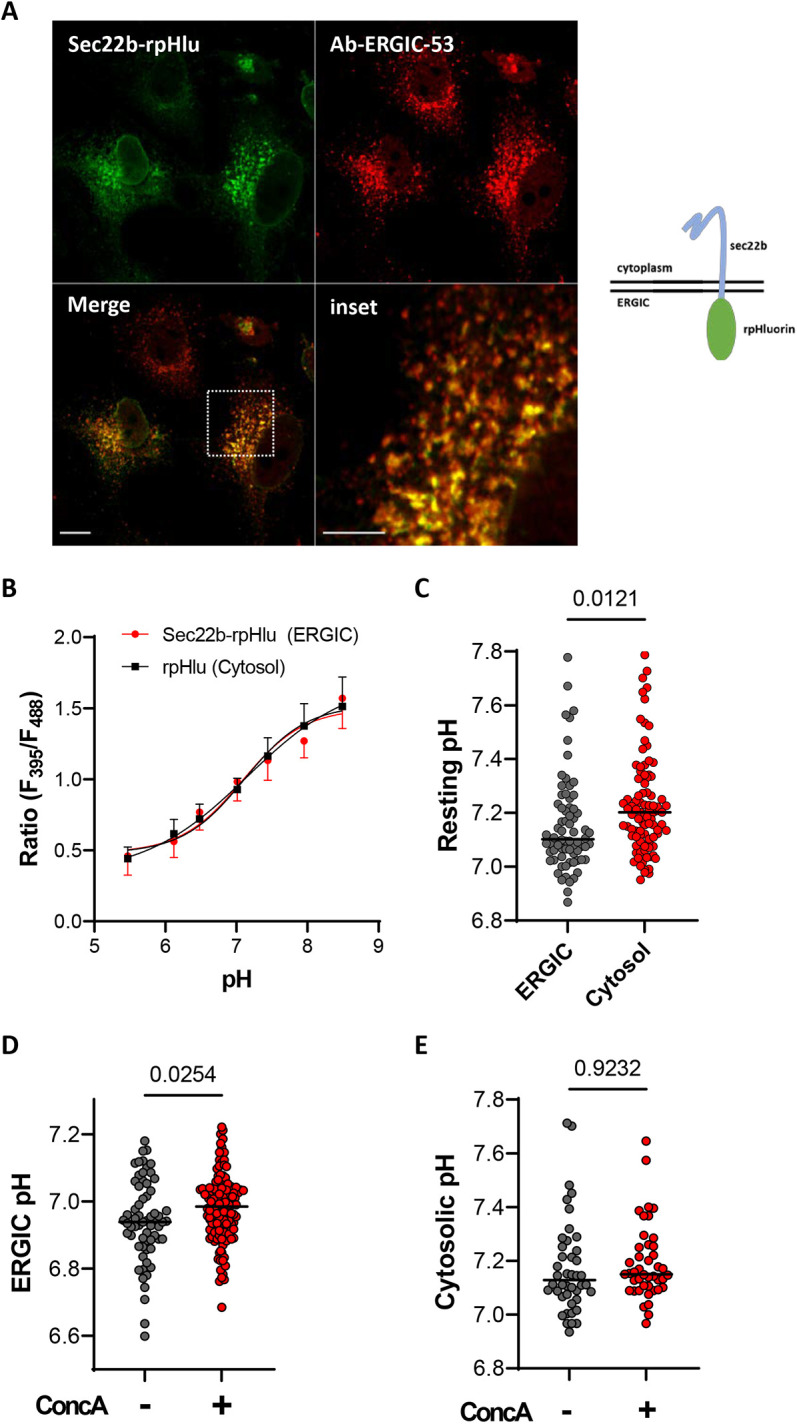
**Recording the pH of the ERGIC lumen in Vero E6 cells.** (A) Representative fluorescence images from four repeats of Vero E6 cells transiently expressing Sec22b–rpHlu (green) and stained with an anti-ERGIC53 antibody (red). Scale bars: 10 µm. The diagram to the right shows the features of the Sec22b–rpHlu construct used to measure ERGIC pH. (B) *In situ* pH titration of rpHluorin and Sec22b–rpHlu fluorescence ratio on an automated cell imaging system (λex=395/488, λem=510). Data are mean±s.d. Each dot is the average of 40–50 cells from two experiments, each with 15–20 image fields. Lines are sigmoidal fits of the data. (C) Calibrated pH values reported by the rpHluorin probe in the ERGIC and cytosol of Vero E6 cells. *n*=72 and 96 cells, respectively, from two independent experiments; lines show median values. (D,E) Effect of concanamycin A (ConcA, 1 µM, 10 min) on the resting ERGIC and cytosolic pH of Vero E6 cells. *n*=64 and 131 (ERGIC) and 44 and 43 cells (cytosol, red circles) for without and with ConcA, respectively, from two independent experiments; lines show median values. All *P*-values calculated by two-tailed unpaired Student's *t*-test.

To assess whether viral infection with SARS-Cov2 alters the pH of the ERGIC lumen, we fused the ERGIC transmembrane protein Sec22b to the ratiometic pH reporter probe pHluorin (Sec22b–rpHlu) (Fig. 1A). When expressed in Vero E6 cells, Sec22b–rpHlu decorated punctate perinuclear structures that colocalized extensively with ERGIC-53 (also known as LMAN1) immunoreactivity (Hauri et al., 2000), validating the proper targeting of the pH probe (Fig. 1A). Calibration on a high-resolution fluorescence microscope showed that the fluorescence ratio of Sec22b–rpHlu increased 3.4-fold in Vero cells as pH was equilibrated from 5.5 to 8.0 with ionophores, with a pKa of 6.81 being well resolved on a log-log pH titration fit (Fig. S1A). Nearly identical calibration curves were obtained in cells transfected with the cytosolic rpHlu and the ERGIC Sec22b–rpHlu, grown on 96-wells plates and imaged on an automated microscope placed in a biosafety level 3 (BSL3) laboratory (Fig. 1B; Fig. S1). The calculated ERGIC pH of cells imaged within the BSL3 isolator was slightly more acidic than their cytosolic pH (pH_cyto_=7.27±0.03 versus pH_ERGIC_=7.16±0.02, mean±s.e.m.; Fig. 1C). Inhibition of the vacuolar H^+^-ATPase with concanamycin A (ConcA, 1 µM for 10 min) increased ERGIC pH by 0.04 units without affecting the cytosolic pH (Fig. 1D,E), indicating that the mildly acidic pH of the ERGIC reflects proton pumping by V-ATPases. These data validate Sec22b–rpHlu as a reliable quantitative reporter of the ERGIC luminal pH and reveals that this compartment is acidified by vacuolar proton ATPases.

### SARS-Cov2 infection prevents the acidification of the ERGIC

**Fig. 2.**
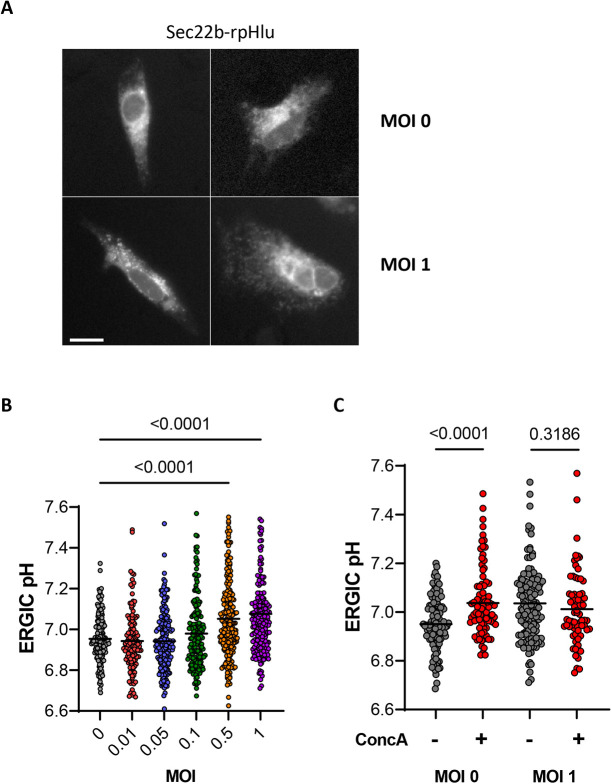
**Effect of SARS-CoV-2 infection on the ERGIC pH of Vero E6 cells.** (A) Representative fluorescence images (λex=488, λex=510 nm) of Vero E6 cells expressing Sec22b–rpHlu and infected with 0 or 1 MOIs of the delta SARS-CoV2 virus. Scale bar: 10 µm. (B) pH values reported by the ERGIC probe in Vero E6 cells infected with different MOIs of the Delta SARS-CoV2 virus. *n*=179, 126, 182, 181, 219 and 211 cells (from 0 to 1 MOI) from three independent experiments performed in duplicates; lines show median values. *P*-values calculated by ordinary one-way ANOVA with Tukey's multiple comparisons test. (C) ERGIC pH of Vero E6 cells infected with 0 and 1 MOIs of the delta SARS-CoV2 virus and treated or not with concanamycin A (ConcA, 1 µM, 10 min). *n*=115, 91 for MOI 0, and 131, 72 for MOI 1 cells from two independent experiments; lines show median values. *P*-values calculated by two-tailed unpaired Student's *t*-test.

Next, we measured pH_cyto_ and pH_ERGIC_ in Vero E6 cells infected with increasing multiplicity of infection (MOI) amounts of the delta SARS-CoV2 virus for 24 h. Viral infection did not alter the expression levels and subcellular distribution of the Sec22b–rpHluorin probe (Fig. 2A). The pH_cyto_ was stable as the viral load increased, ranging from 7.18±0.02 at MOI 0 to 7.25±0.03 at MOI 1 (mean±s.e.m.; Fig. S2A) and remained insensitive to ConcA (Fig. S2B). In contrast, pH_ERGIC_ was significantly higher at MOIs above 0.5, increasing from 6.95±0.01 at MOI 0 to 7.08±0.02 at MOI 1 (Fig. 2B), and the ERGIC alkalinization evoked by ConcA was not observed in infected cells (Fig. 2C). These data indicate that infection with the SARS-Cov2 delta virus dissipates the acidic ERGIC pH maintained by V-ATPases.

### SARS-Cov2 infection mitigates lysosomal acidification

**Fig. 3.**
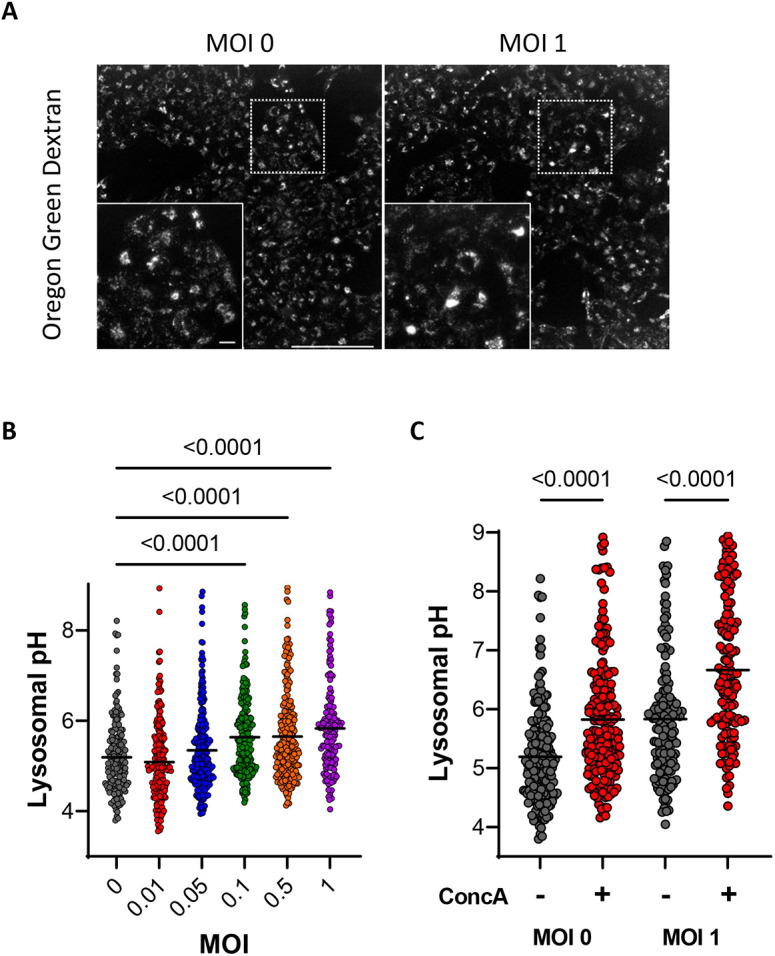
**Effect of SARS-CoV-2 infection on the lysosomal pH of Vero E6 cells.** (A) Representative fluorescence images (λex=488, λem=510) of Vero E6 cells loaded overnight with OGDx and infected with 0 or 1 MOIs of the delta SARS-CoV2 virus. Scale bars: 200 µm (main image); 20 µm (inset). (B) Lysosomal pH of Vero E6 cells infected with different MOIs of the delta SARS-CoV2 virus. *n*=200, 239, 240, 236, 233 and 157 cells (from 0 to 1 MOI) from two independent experiments; lines show median values. *P*-values calculated by ordinary one-way ANOVA with Tukey's multiple comparisons test. (C) Lysosomal pH of Vero E6 cells infected with 0 and 1 MOIs of the delta SARS-CoV2 virus, treated or not with concanamycin A (ConcA, 1 µM, 10 min). *n*=200, 195 for MOI 0, and 157, 140 for MOI 1 cells from two independent experiments; lines show median values. *P*-values calculated by two-tailed unpaired Student's *t*-test.

Assembled β-coronaviruses exit through lysosomal exocytosis, and SARS-Cov2 infection has been reported to prevent lysosomal acidification (Cabrera-Garcia et al., 2021; Ghosh et al., 2020). We therefore measured the lysosomal pH (pH_lyso_) by exposing Vero E6 cells overnight to dextran particles labeled with Oregon Green (OGDx). Calibration indicated that OGDx fluorescence increased 4-fold in the pH range 4 to 6 (Fig. S3). Infection with increasing MOIs of the delta SARS-CoV2 virus for 24 h did not alter the OGDx loading pattern but increased OGDx fluorescence intensity (Fig. 3A), corresponding to an increase in pH_lyso_ from 5.19±0.06 at MOI 0 to 5.83±0.08 at MOI1 (mean±s.e.m.; Fig. 3B). Addition of ConcA (1 µM for 10 min) increased pH_lyso_ by 0.63 and 0.83 pH units at MOI 0 and MOI 1, respectively (Fig. 3C). These data indicate that infection with the delta SARS-Cov2 variant also mitigates the acidification of lysosomes. Unexpectedly, we observed that bafilomycin alkalinized lysosomes very slowly in Vero E6 cells compared to what was seen in HeLa cells, whereas the protonophore CCCP caused a rapid alkalinization in both cell lines (Fig. S3B). Further experiments indicated that a 30 min incubation with bafilomycin was required to raise pH_lyso_ to pH ∼7.0 in Vero E6 cells (Fig. S3C). Vero E6 cells thus appear to have a low lysosomal proton leak and require a long exposure to V-ATPase inhibitors.

### The envelope protein of SARS-CoV-2 accumulates in the ERGIC when expressed in Vero E6 cells

**Fig. 4.**
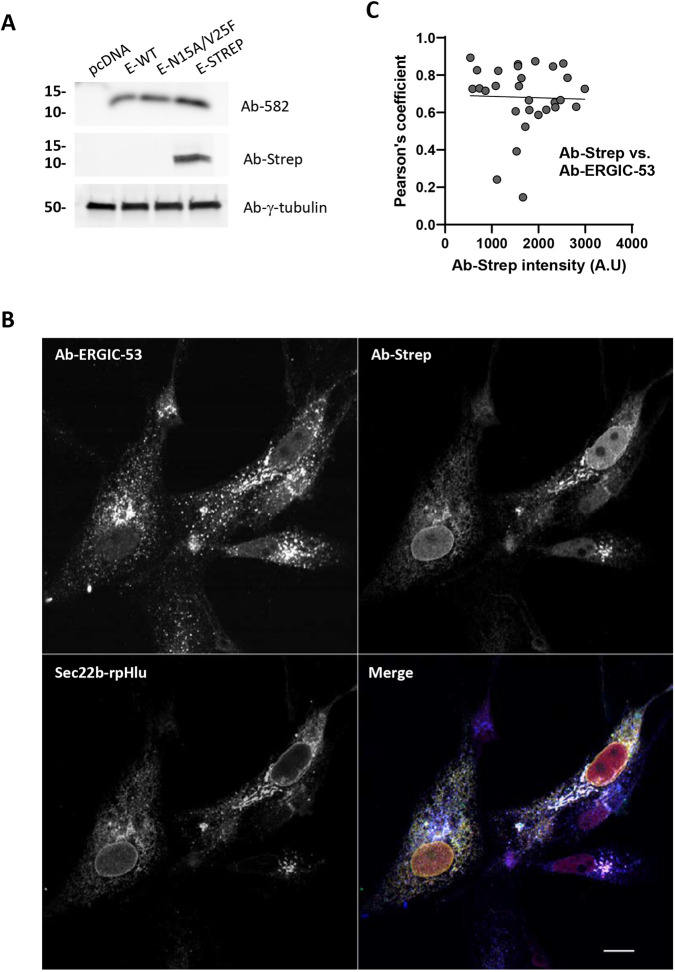
**Expression of the envelope protein of SARS-CoV-2 in Vero E6 cells.** (A) Western blots of whole-cell lysates from Vero E6 cells transiently expressing the empty vector (pcDNA), the native SARS-CoV-2 E protein (E-WT), the N15A/V25F SARS-CoV-2 E double mutant (E-N15A/V25F), and the streptavidin-tagged SARS-CoV-2 E protein (E-Strep). Blots were revealed with a recombinant antibody raised against the native SARS-CoV-2 E protein (Rb-582, top), an anti-streptavidin antibody (middle) or with anti-γ-tubulin antibody as a loading control (bottom). Image representative of two repeats. (B) Fluorescence images from Vero E6 cells transiently expressing Sec22b–rpHlu (green) together with Strep-tagged SARS-CoV2 E protein (red, streptavidin immunoreactivity), stained with an anti-ERGIC53 antibody (blue). Images representative of three independent experiments. Scale bar: 10 µm. (C) Pearson's correlation coefficient between E-Strep and ERGIC-53 immunoreactivities as a function of E-Strep expression levels. *n*=30 cells. A.U, arbitrary units.

**Fig. 5.**
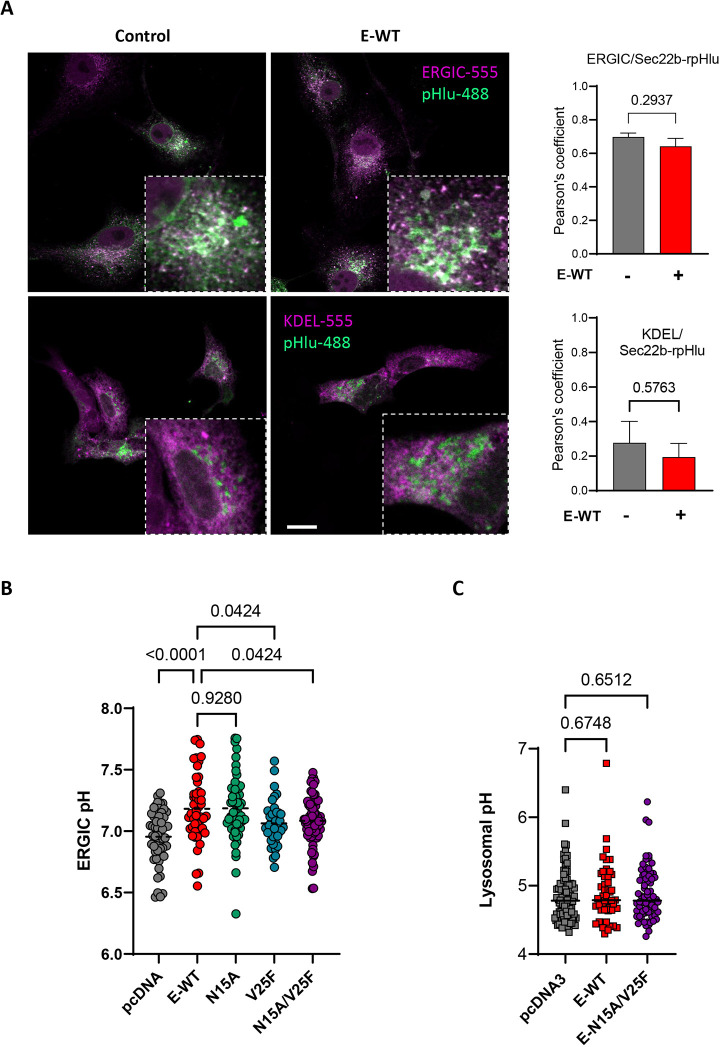
**Effect of SARS-CoV-2 E on the cytosolic and organelle pH.** (A) Confocal micrographs of Vero E6 cells expressing Sec22b–rpHlu (pHlu-488) alone (left images) or together with E-WT protein (right images), stained for ERGIC-53 (top row) or co-expressing KDEL-RFP (bottom row). Scale bar: 20 µm. Graphs show Pearson's correlation coefficients. Data are mean±s.e.m. *n*=16, 16 (ERGIC with and without E-WT) and 9, 10 (KDEL with and without E-WT) cells. *P*-values calculated by two-tailed unpaired *t*-test. (B) ERGIC pH of Vero E6 cells transiently expressing the indicated constructs; lines show median values. *n*=55, 50, 58, 40, 70 individual cells per condition from left to right in three independent experiments. *P*-values calculated by ordinary one-way ANOVA with Holm–Šídák's multiple comparisons test. (C) Lysosomal pH of Vero E6 cells transiently expressing the indicated constructs; lines show median values. *n*=94, 48, 70 individual cells per condition from left to right in two independent experiments. *P*-values calculated by ordinary one-way ANOVA with Dunnett's multiple comparisons test.

To test whether the pH-dissipating effects of the SARS-CoV-2 virus reflect the activity of its E protein in organelles, we transiently expressed plasmids coding for the native and epitope-tagged E protein in mammalian Vero E6 cells. Expression of wild-type SARS-CoV-2 E in Vero E6 cells was confirmed by the detection of a ∼15 kDa band on western blots with an in-house generated recombinant antibody directed against the native protein (Fig. 4A). A band of similar size was detected in cells expressing E mutated at residues Asn15 and Val25 within the putative pore domain, and with antibodies against the streptavidin epitope (WSHPQFEK) added to the C-terminus of the protein (Fig. 4A), confirming expression of the recombinant proteins. Streptavidin immunoreactivity was detected in Vero E6 cells expressing Strep-tagged E in structures overlapping with ERGIC-53 immunoreactivity and with co-expressed Sec22b–rpHlu (Fig. 4B). The Strep-tagged E colocalized extensively with GFP–ERGIC-53 and the expression levels of the E protein itself did not affect its colocalization with the ERGIC marker (Fig. 4C). To assess the cellular toxicity associated with the expression of E, we measured cell death and ER stress in Vero E6 cells. Short-term (24 h) expression of E did not induce apoptosis or ER stress, whereas longer expression (72 h) slightly reduced basal UPRE promoter activity, without altering the ER stress response induced by tunicamycin (Fig. S4). E protein expression thus causes a mild reduction in basal UPRE activity but does not sensitize Vero E6 cells to ER stress. It is possible that co-expression of the viral protein might alter the ER versus ERGIC distribution of Sec22b–rpHlu, skewing the pH measurements towards the more alkaline pH of the ER. To rule out this possibility, we quantified the colocalization of Sec22b–rpHlu with the ERGIC and ER markers in cells expressing or not wild-type E (E-WT). The fraction of the signal colocalizing with RFP–KDEL was low (∼20%), with the probe colocalizing preferentially with ERGIC-53 immunoreactivity (≈60%) regardless of viral protein co-expression (Fig. 5A; Fig. S5). These data indicate that the ectopically expressed viral E protein accumulates preferentially in the ERGIC without causing acute toxicity or altering the subcellular localization of Sec22b–rpHlu.

### The envelope protein of SARS-CoV-2, but not its V25F or N15A/V25F mutants, dissipates the ERGIC pH

Next, we measured the impact of E protein expression on the ERGIC pH. Acute expression of E-WT increased the resting pH_ERGIC_ of Vero E6 cells from 6.95±0.03 to 7.18±0.04 (mean±s.e.m.; Fig. 5B). We then analyzed the effect of mutations of residues N15 and V25 predicted to face the channel pore (Mandala et al., 2020). Expression of N15A increased pH_ERGIC_ to 7.19±0.03, similar to the E-WT protein, whereas expression of V25F or of the double N15A/V25F mutant did not alter pH_ERGIC_ (Fig. 5B). Unexpectedly, pH_lyso_, as determined by OGDx ratio fluorescence imaging, was not altered by the expression of the E-WT protein or by the double N15A/V25F mutant (Fig. 5C). This indicates that the expression of the envelope protein of SARS-CoV-2 dissipates the ERGIC pH without affecting the acidic pH of lysosomes. The viroporin effect of the envelope protein in the ERGIC is prevented by mutation of Val25 but not of Asn15, indicating that the Val25 residue is critical for proton permeation.

## DISCUSSION

In this study, we report that infection of Vero E6 cells with the SARS-COV-2 delta virus alkalinizes the ERGIC and lysosomes and link this pH alteration to the ion channel function of the viral envelope protein in the ERGIC. Using the ratiometric pHluorin fused to Sec22b, we provide the first direct quantitative measurements of the pH within the lumen of the ERGIC. We found that the ERGIC pH of Vero E6 cells is ∼0.2 pH units more acidic than the cytosolic pH, measured with the same genetic indicator, due to the activity of vacuolar H^+^-ATPases sensitive to bafilomycin and concanamycin. SARS-COV-2 infection, at MOI higher than 0.1, dissipated the acidic pH of the ERGIC and lysosomes, mimicking the effects of V-ATPases inhibitors. In non-infected cells, enforced expression of the SARS-COV-2 E protein dissipated ERGIC pH but did not alter lysosomal pH. This effect was also observed with the N15A mutant of E but not with the V25F or N15A/V25F mutants. These data establish that E acts as a proton channel within the ERGIC, with Val25 critical for proton permeation, and that the SARS-COV-2 virus exploits this viroporin activity to alter the pH of intracellular organelles.

Recent electrophysiological recordings of HEK-293 cells and *Xenopus* oocytes expressing SARS-COV-2 E targeted to the plasma membrane reported large monovalent cations currents that became inward rectifying as the pH was decreased from 8 to 6 in HEK-293 cells (Cabrera-Garcia et al., 2021). This indicates that the E protein forms a cation channel that is activated at luminal acidic pH, as previously reported for other coronaviruses (Cabrera-Garcia et al., 2021; Mandala et al., 2020). These authors further showed that SARS-COV-2 E fused to mKate accumulates in perinuclear structures decorated by an anti-ERGIC-53 antibody and decreases the fluorescence of a membrane-permeant pH-sensitive dye (Lysosensor DND-189, pKa=5.2) in NIH-3T3 cells (Cabrera-Garcia et al., 2021). This indicates that enforced E expression increases the luminal pH of acidic organelles, an effect previously reported for the viroporins of other coronaviruses (Westerbeck and Machamer, 2019).

We confirm here that the SARS-COV-2 E protein accumulates in the ERGIC when ectopically expressed in mammalian cells. In Vero E6 cells, the E protein colocalized extensively with the mannose-specific membrane lectin ERGIC-53, an established ERGIC marker, and with rpHluorin fused to the transmembrane domain of the ERGIC-resident SNARE Sec22b. The epitope-tagged SARS-CoV-2 E protein was detected predominantly in the ERGIC with a minor fraction in the ER. E expression did not impact organelle appearance and did not induce ER stress within 72 h. Enforced E expression, on the other hand, increased the luminal pH of the ERGIC by 0.2 pH units in Vero E6 cells without altering the pH of lysosomes, as determined by measuring internalized OGDx. ERGIC alkalinization was not observed in cells expressing the V25F mutant or the double N15A/V25F mutant but persisted in cells expressing the single N15A mutant. These residues are located within the predicted pore domain, linking the alkalinization to the proton channel function of E. Both Asn15 and Val25 are pore-facing residues in the single pentamer model derived from NMR structures of the envelope protein reconstituted in lipid bilayers (Mandala et al., 2020). Val25 forms an interhelical contact with Phe20 in conditions that promote channel opening (Medeiros-Silva et al., 2022). Other studies reported an interhelical orientation for the side chain of Val25 (Surya et al., 2018) with the aromatic residue Phe26 pointing inward to constrict the pore (Mehregan et al., 2022). Our functional data indicate that Val25 but not Asn15 mutation impacts the proton channel function of E. Val25 mutations might disrupt an aromatic gating ring, shifting the channel to the closed state, as predicted from NMR studies (Medeiros-Silva et al., 2022). Importantly, a similar viroporin activity was detected in cells infected with a replicating SARS-COV-2 delta virus. Viral infection alkalinized the ERGIC and lysosomes, mimicking the effects of V-ATPases inhibitors. The simplest explanation for the ERGIC alkalinization occurring in infected cells is therefore that the viral envelope protein acts as a viroporin in this organelle. The lysosomal alkalinization observed in infected cells was not recapitulated by E protein expression, suggesting that the envelope protein might not reach lysosomes when overexpressed, consistent with its extensive colocalization with the ERGIC marker. Viral infection might reprogram the cellular secretory pathway to target the viral particles to lysosomes for egress, enabling viroporin activity in this highly acidic compartment.

Our report that SARS-CoV-2 infection alters the ERGIC and lysosomal pH of mammalian cells has implications for virus fitness and pathogenicity. The SARS-COV-1 E protein accumulates in the ER and Golgi, and its viroporin activity in these organelles is thought to facilitate virus propagation and pathogenicity (DeDiego et al., 2007; Nieto-Torres et al., 2014). We show that the proton channel function of SARS-COV-2 E counteracts ERGIC acidification by vacuolar ATPases. Mitigating ERGIC and lysosome acidification might protect newly formed virions from a toxic acidic environment. An acidic pH is important for dissociation of cargo from sorting lectins such as ERGIC-53 (Appenzeller-Herzog et al., 2004) and SARS-CoV-2 might exploit this mechanism to promote viral particle assembly by relying on the viroporin activity of E to dissipate the pH gradient. Preventing organelle acidification is also expected to disrupt secretory cargo relying on a pH-sensitive dissociation mechanism, like procathepsin. The ion channel function of the SARS-CoV-2 E protein therefore likely contributes to SARS-CoV-2 propagation and pathogenicity, making this viroporin an attractive drug target.

In summary, we show here that SARS-CoV-2 infection prevents ERGIC and lysosomes acidification in infected cells and link the ERGIC pH deregulation to the viroporin activity of the viral envelope protein. The channel function of E likely contributes to SARS-CoV-2 fitness and pathogenicity by alkalinizing organelles. Compounds inhibiting this viroporin could provide new antiviral drugs targeting an essential viral function conserved among coronaviruses.

## MATERIALS AND METHODS

### Reagents

Concanamycin A was purchased from Enzo Life Sciences (AXL-380-034-C100), and bafilomycin A1 from Sigma (B1793). The mouse anti SARS-CoV-2 E protein nanobody Rb-582 was developed by the Geneva antibody facility as described at Marchetti et al. (2020), the mouse anti-Strep tag antibody was purchased from BioLegend (688202), the mouse anti-γ-tubulin antibody from Thermo Fisher Scientific (MA1-850) and the rabbit anti-ERGIC-53 antibody from Merck (E1031). Secondary antibodies for immunofluorescence were goat anti-mouse-IgG (H+L), Alexa Fluor® 647 conjugate (ref. A21235) and goat anti-rabbit-IgG (H+L), Alexa Fluor® 555 conjugate (ref. A21428) both Thermo Fisher Scientific. Secondary antibodies for western blotting were goat anti-mouse IgG (H+L)-HRP conjugate (ref: 1706516) and goat anti-rabbit IgG (H+L)-HRP conjugate (ref. 1706515) both from Bio-Rad. The SARS-CoV-2 E-protein wild-type, N15A, V25F and N15A/V25F cDNA sequences were ordered as synthetic plasmids with flanking EcoRI and XhoI cut sites from GeneArt. The cDNA sequences were cloned into pcDNA (a kind gift from Dr Stephane Konig, UNIGE, Switzerland) using EcoRI and XhoI enzymatic digestion. The pLVX-EF1a-nCOV2019-E-2xstrepIRES-Puro carrying the SARS-CoV-2 E protein with two Strep tags was obtained from Addgene (#141385; Gordon et al., 2020). The pENTR1a-ER-RFP (KDEL-tagRFP) was obtained from GenScript (no. Sc 1622). The pCMV-rpHluorin-N1 was a kind gift from Dr Thierry Galli (INSERM, Paris, France). The pCMV-sec22b-rpHluorin was generated by cloning Sec22b (generated by PCR with flanking NheI and XhoI cut sites) into the multiple cloning sites of pCMV-rpHluorin-N1, using NheI and XhoI enzymatic digestion.

### Cells and transfection

Vero-E6 cells were a kind gift from Pre Caroline Tapparel (UNIGE, Geneva, Switzerland) and Calu-3 cells were a kind gift from Dr Karl-Heinz Krause (UNIGE, Geneva, Switzerland). Vero E6 cells were cultured in Dulbecco's minimal essential medium (DMEM) supplemented with 10% FBS and Pen/Strep and maintained at 37°C and 5% CO_2_. Cells were grown to 80% confluency prior to transfection or co-transfection with different plasmids for 24 h using Lipofectamine 2000 (Thermo Fisher Scientific).

### Viral preparation and infection

The delta SARS-CoV2 virus was a kind gift from Dr Isabella Eckerle (University Hospital Geneva, Switzerland). For propagation, the virus was cultured with Calu-3 cells for 72 h prior to collection of media and clarification of cell debris by centrifugation (2000 ***g***). The plaque forming units per volume (pfu/ml) of the virus was 10^7^, determined by Vero E6 cell infection followed by plaque assay in a 24-well plate format, using cells plated to 80–90% in a 24-well plate and were infected with serial dilutions of the virus.

### pH measurements

For cytoplasmic and ERGIC pH recordings, Vero E6 cells were transfected with pCMV-rpHluorin-N1 or pCMV-sec22b-rpHluorin. Cells transiently expressing or not the envelope protein were alternatively excited for 100 ms with ET380x and ET490/20 filters and the rpHluorin ratio fluorescence imaged with a 525/50 band pass filter (Chroma) at 37°C on a Nikon Eclipse Ti inverted microscope equipped with a 60× Plan Apo 1.30 NA objective, a Sutter Lambda XL lamp and a bipolar temperature control stage heater (Harvard Apparatus), controlled by Visiview software (Visitron Systems). Cells infected with the virus were imaged inside a BSL3 lab on a PicoXpress microscope (Molecular Devices) on 96-well plates alternatively excited for 500 ms through the FITC 445-485/509-539 filter cube and 800 ms through the customized F49-395 395/25ET Bandpass, F48-425 Beamsplitter T 425 LPXR, F47-525 525/50 ET Bandpass filter cube on the HC PL FLUOTAR 20×/0.40 objective.

For lysosomal pH, Vero E6 cells were loaded overnight with Oregon Green™ 488, 10,000MW dextran (OGDx) (D7171, Thermo Fisher Scientific) and prepared and imaged as previously described (Pihan et al., 2021). Cells infected with the virus were imaged with the PicoXpress in the BSL3 laboratory, using a single excitation for 1000 ms through the FITC 445-485/509-539 filter cube.

pH calibration was performed using nigericin (5 μg/ml) and monensin (5 μM) in solutions containing 125 mM KCl, 20 mM NaCl, 0.5 mM MgCl_2_ and 0.2 mM EGTA, with HEPES (pH 7.0–7.5), MES (pH 5.5–6.5) or acetic acid (pH 4–4.5) or citric acid (pH 3–3.5) as previously described (Nunes et al., 2015; Pihan et al., 2021). The cells were incubated with each calibration solution for 3 min before imaging. For each experiment, a five-point calibration curve was fitted to a variable slope sigmoid equation using GraphPad Prism. Cells were imaged in modified Ringer's buffer or in Vero E6 culture medium supplemented with 25 mM HEPES.

### Luciferase experiments

The luciferase assay was performed by co-transfecting luciferase response elements with *Renilla* (Carreras-Sureda et al., 2021) and empty vector or E protein. At 48 h after transfection cells were treated or not with tunicamycin for 24 h (100 ng/ml; Sigma, SML1287) and measured for luciferase activity using a Promega dual luciferase reporter kit. ERSE and UPRE reporters were described in Yoshida et al. (2003) and AARe element-luciferase in Bruhat et al. (2000).

### Western blotting

Following transfection, cells were harvested with RIPA lysis buffer (Sigma; R0278) containing protease inhibitor (Sigamafast^TM^ protease inhibitor cocktail tablets, EDTA-free) for 30 min on ice. Cell lysates were centrifuged at 11,200 ***g*** for 10 min and the supernatant was diluted with 4X NuPAGE LDS Sample Buffer (Thermo Fisher Scientific; NP0007). Samples were subjected to electrophoresis through 4-20% mini-protean^®^ TGX™ precast gels (Bio-Rad; 4561095), membrane transfer and immunoblot analysis. Immunoblots were probed with mouse anti SARS-CoV-2 E protein nanobody (1:250), mouse anti STREP-Tag (1:1000) and mouse anti γ-tubulin (1:2000).

### Immunofluorescence

Immunofluorescence was performed in Vero E6 cells co-transfected with the indicated constructs. After 24 h of transfection, cells were fixed (4% PFA) for 20 min at room temperature, then permeabilized (0.5% BSA in PBS plus 0.5% Triton X-100) for 10 min at room temperature and blocked (2% BSA in PBS) for 1 h at room temperature. Cells were then incubated with primary antibodies overnight at 4°C in a wet chamber and then incubated with the corresponding secondary antibodies coupled to fluorochromes (1:1000) for 1 h at room temperature. Images were obtained on a LSM700 Nikon microscope.

### Image analysis and statistics

Image analysis was performed with ImageJ. Data analysis was performed with GraphPad Prism 8. Student's *t*-test and ANOVA statistical analysis were used where appropriate. *P*-values are indicated directly on the graphs.

## Supplementary Material

10.1242/joces.260685_sup1 Supplementary information
