# Supplementary material for: SARS-CoV-2 infection alkalinizes the ERGIC and lysosomes through the viroporin activity of the viral envelope protein
Source: J Cell Sci. 2023 Mar 24;136(6):jcs260685. doi: 10.1242/jcs.260685 (PMC10112968; doi:10.1242/jcs.260685)
Supplement: Supplementary information [file joces-136-260685-s1.pdf]

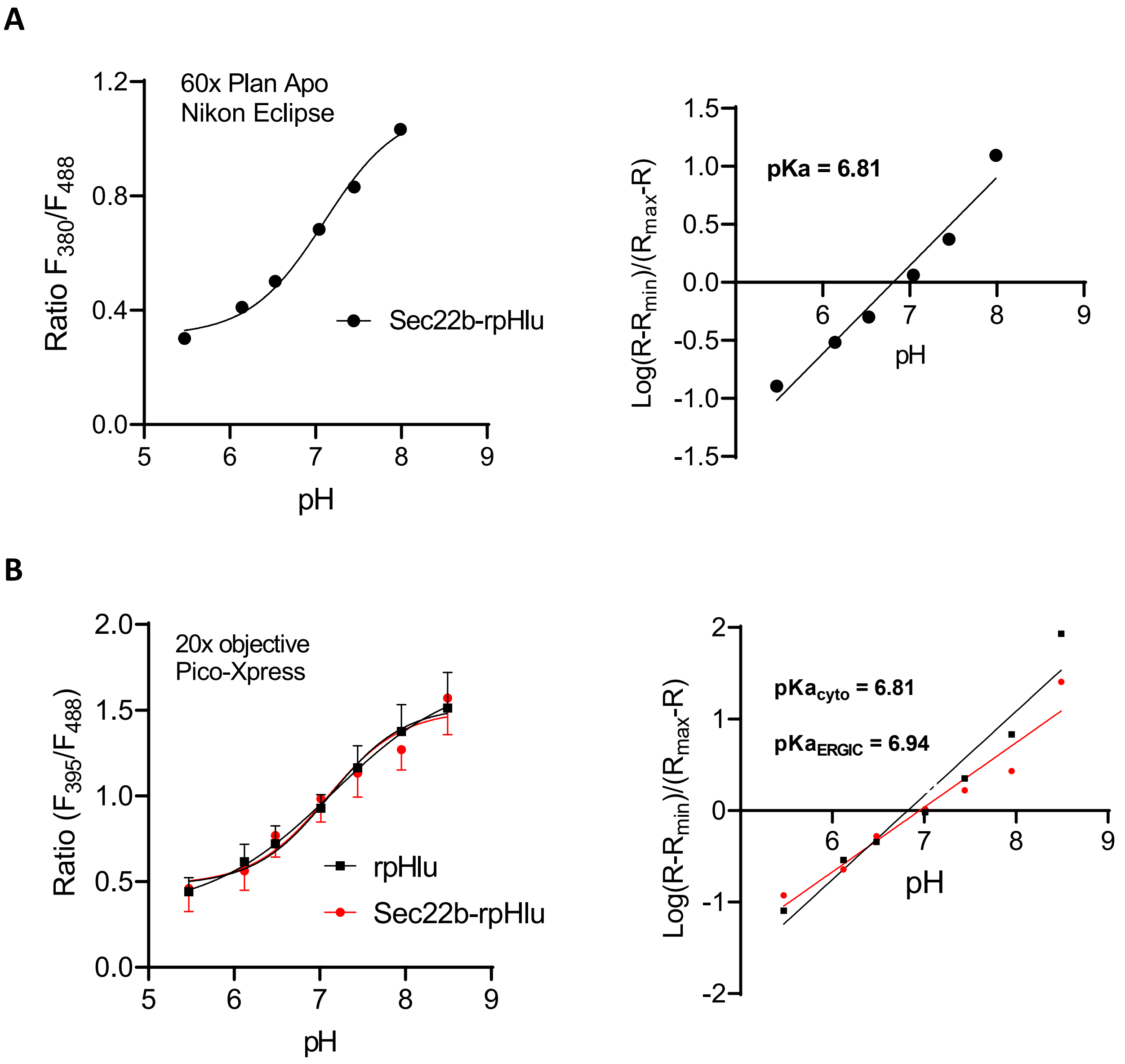

Fig. S1. Calibration of cytosolic and ERGIC-targeted rpHluorin

(A) Left: *In-situ* pH titration of sec22b-rpHluorin fluorescence ratio on a high-resolution fluorescence microscope (λex=380/488, λem=510). Each dot is the average of 40-50 cells from 2 experiments, each with 15-20 image fields. Line is a sigmoidal fit of the data. Right: log-log display of the pH titration curve. Line is a linear fit of the data, crossing the x axis at the probe's pKa. (B) *In-situ* pH titration of rpHluorin and sec22b-rpHluorin fluorescence ratio on an automated cell imaging system (λex=395/488, λem=510). Data from Fig. 1B. Right: log-log display of the pH titration curves with linear fits that cross the x axis at nearly identical values corresponding to the probe's pKa.

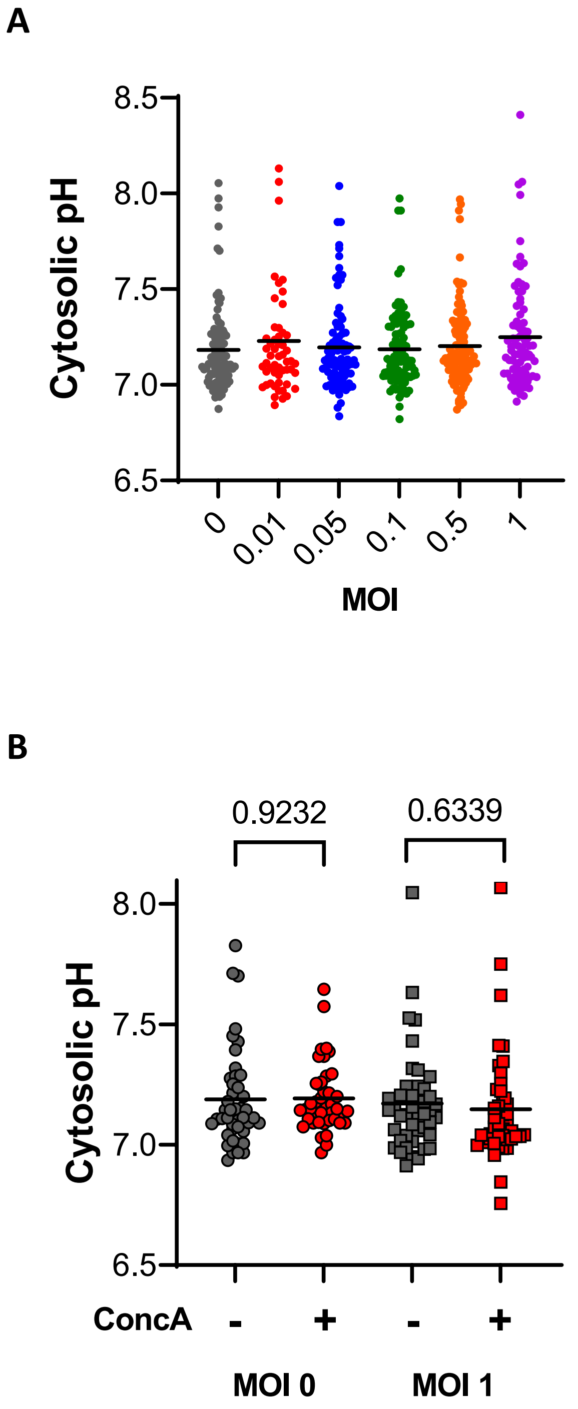

Journal of Cell Science • Supplementary information

Fig. S2. Effect of SARS-CoV-2 infection on the cytosolic pH of Vero E6 cells
(A) Cytosolic pH of Vero E6 cells infected with different MOIs of the Delta SARS-CoV2 virus. N= 94/54/89/96/110/85 individual cells per condition from 3 independent experiments performed in duplicates, lines are median values. No significant variation with ordinary two-way ANOVA. (B) Cytosolic pH of Vero E6 cells infected with 0 and 1 MOIs of the Delta SARS-CoV2 virus and treated or not with ConA (1µM, 10min). N=44/43 (MOI 0, (data from Fig. 1E) and 43/44 (MOI 1) individual cells from 2 independent experiments, lines are median values. Two-tailed unpaired Student's t test.

Journal of Cell Science • Supplementary information

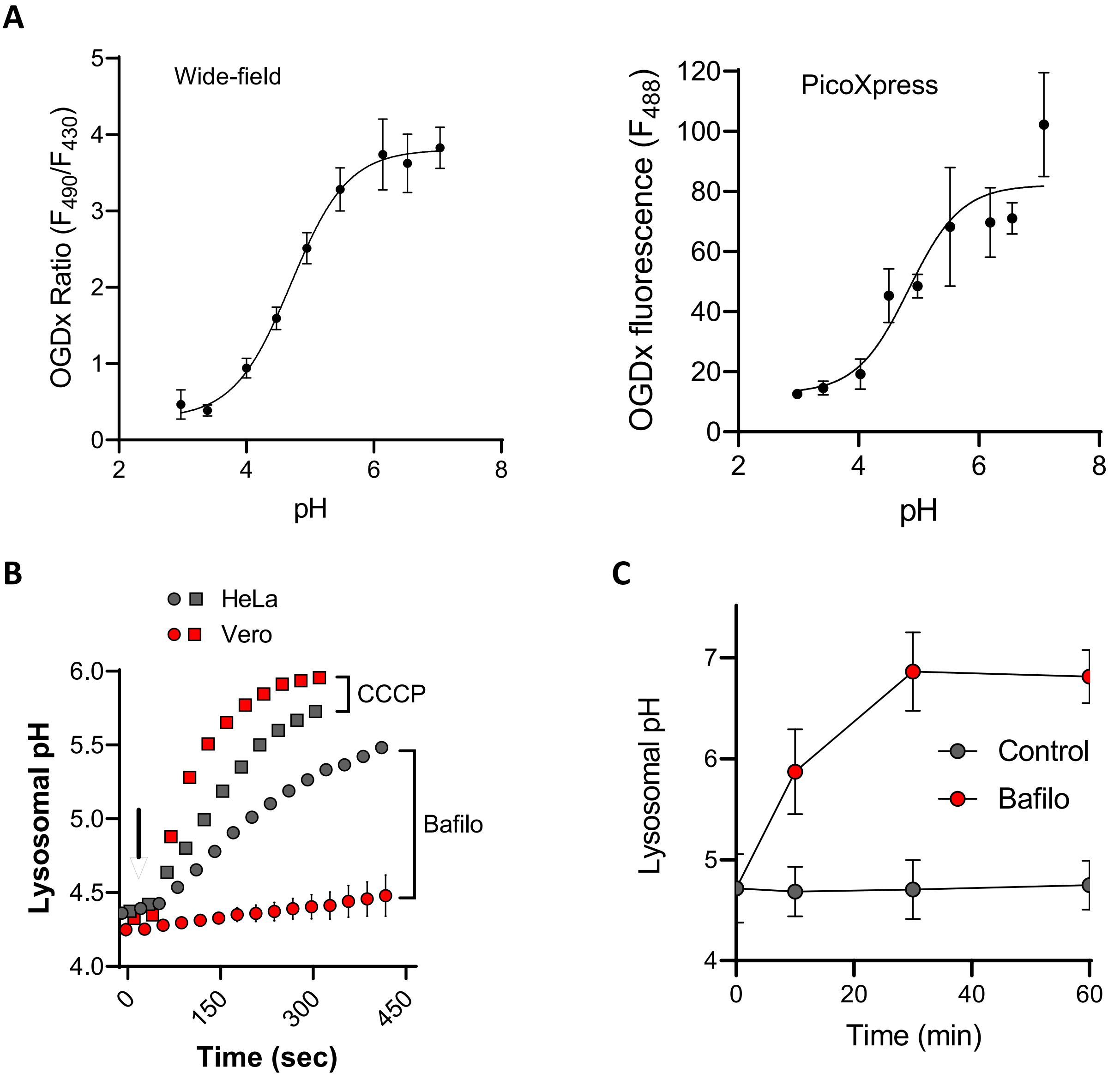

Fig. S3. Calibration of OGDx internalized in lysosomes and effects of V-ATPase inhibitors

(A) *In-situ* pH titration of internalized OGDx-488 ratio fluorescence (λex=430/490, λem=530) on a high-resolution microscope (left) and of OGDx-488 single fluorescence (λex=490, λem=530) on the automated cell imaging system (right). Each dot shows the average fluorescence of 20 cells from 4-5 fields in one of 2 independent measurements. Lines show sigmoidal fits of the data. (B) Effect of acute addition of bafilomycin (10 µM) and CCCP (1 µM) on the lysosomal pH in HeLa cells (gray symbols) and Vero E6 cells (red symbols). (C) Time-course of the lysosomal pH changes in Vero E6 cells treated or not with bafilomycin. Lysosomal pH was measured with OGDx-488 ratio fluorescence. Data are mean±SD of N=203/210/351/206 (Control) and 203/444/237/474 cells (Bafilo) in 2 independent experiments.

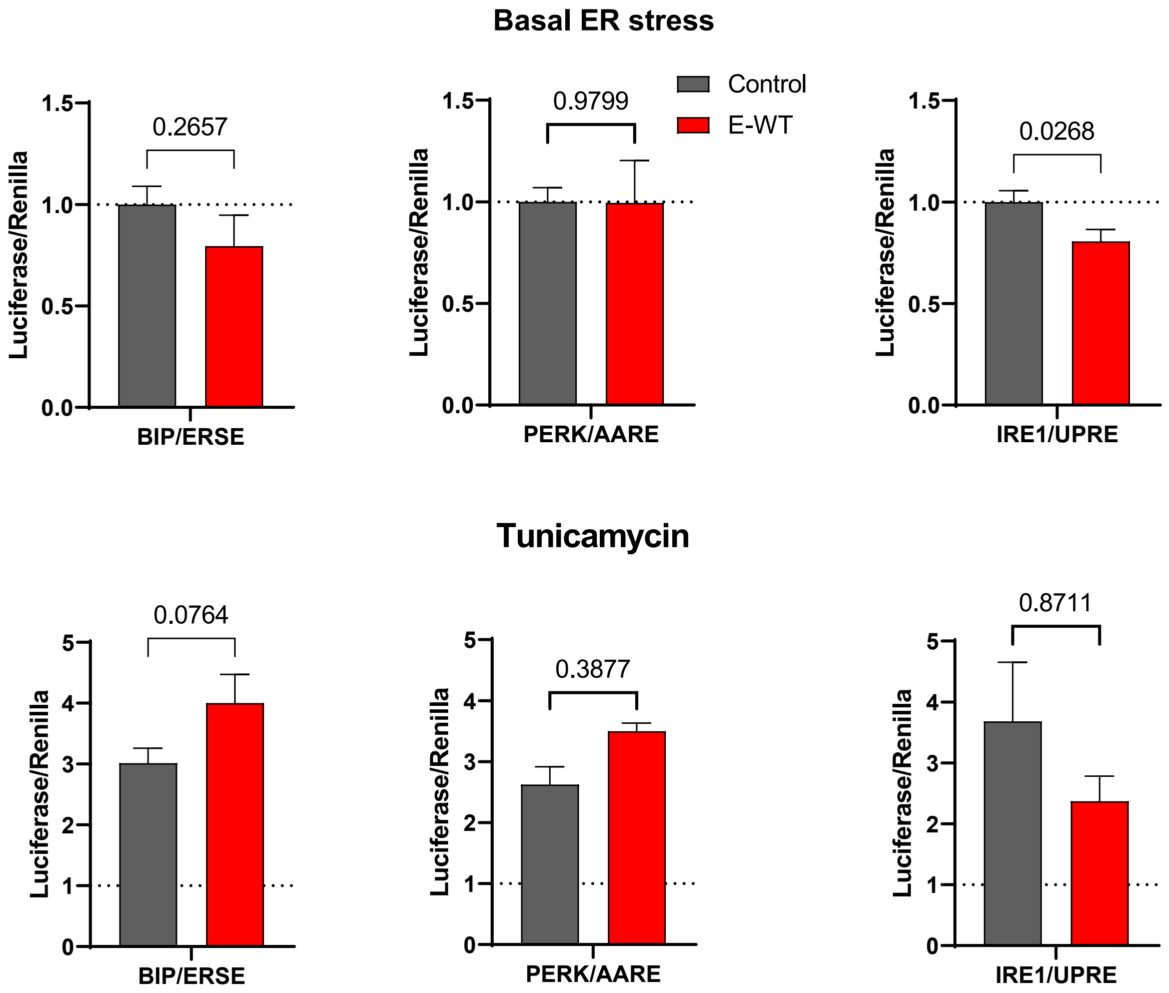

Fig. S4. Expression of SARS-CoV-2 E in HeLa cells.
Top. Basal ER stress levels in Vero E6 cells expressing pcDNA3 or E-WT, measured with ERSE, UPRE, and AARE luciferase reporters. N=10-12 reads from 3 independent experiments. Bottom. ER stress levels in cells expressing the indicated constructs treated with 100ng/ml tunicamycin for 24h. N = 10- 12 reads from 3 different experiments. Two-tailed unpaired Student’s t test.

Journal of Cell Science • Supplementary information

A

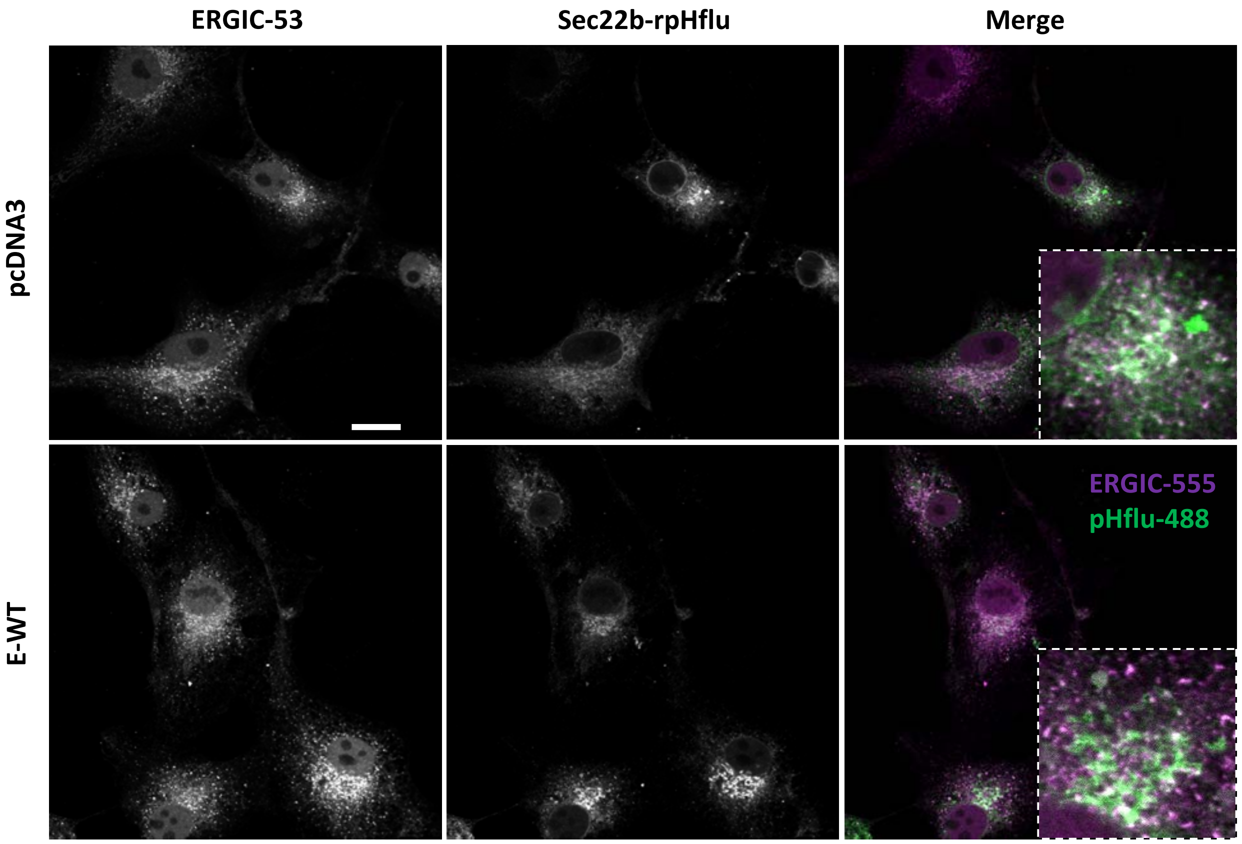

B

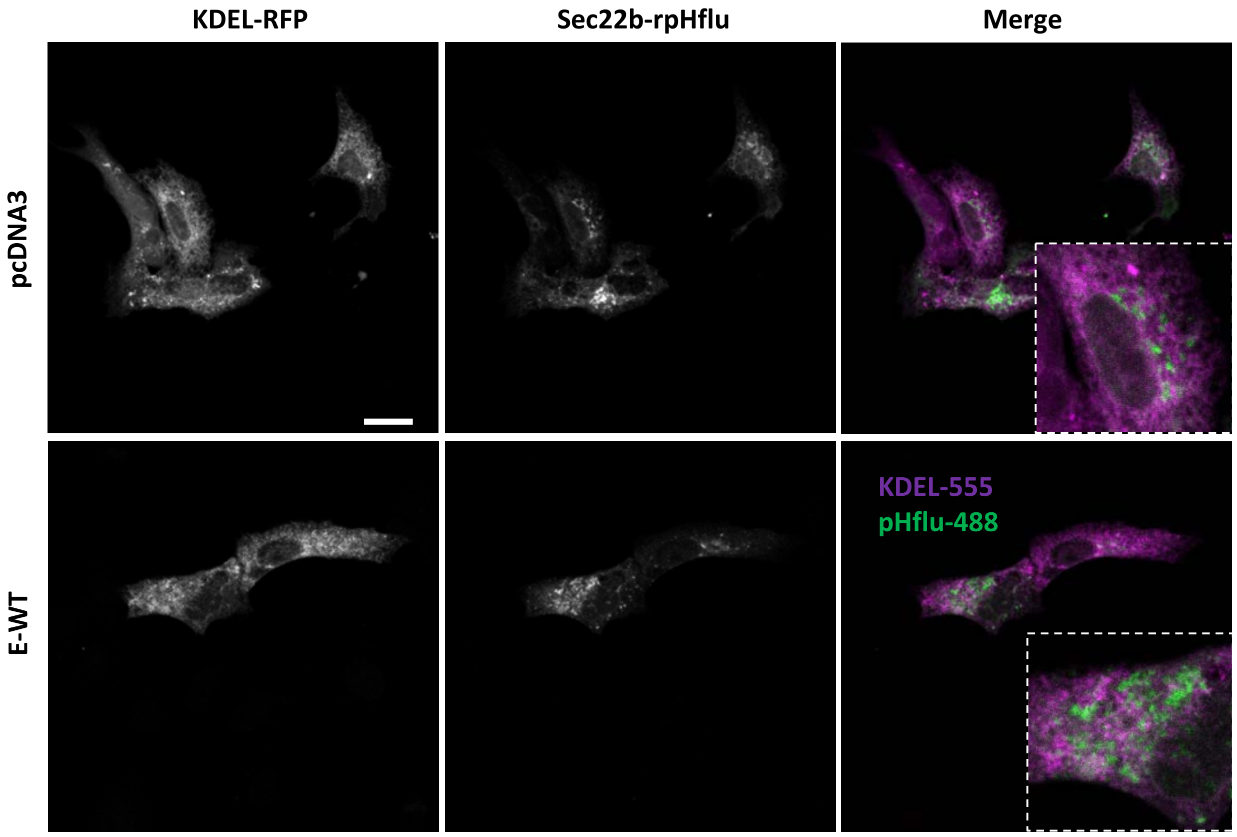

Journal of Cell Science • Supplementary information

Fig. S5. Localization of ectopically expressed SARS-CoV-2 E in Vero E6 cells
Confocal micrographs of Vero E6 cells expressing Sec22b-rpHl alone (top rows) or together with E-WT protein (bottom rows), stained for ERGIC-53 (A) or co-expressing KDEL-RFP (B). Left panels show the fluorescence or the ER and ERGIC markers, middle panels the fluorescence of the pH probe, and right panels the merged images (shown in Fig. 5A). Bar size: 20µm.
