# Supplementary material for: SARS-CoV-2 infection alkalinizes the ERGIC and lysosomes through the viroporin activity of the viral envelope protein
Source: J Cell Sci. 2023 Mar 24;136(6):jcs260685. doi: 10.1242/jcs.260685 (PMC10112968; doi:10.1242/jcs.260685)
Supplement: Supplementary file 1 [file joces-136-260685_review_history.pdf]

Journal of Cell Science

SARS-CoV-2 infection alkalinizes the ERGIC and lysosomes through the viroporin activity of the viral envelope protein

Wen-An Wang, Amado Carreras-Sureda and Nicolas Demaurex
DOI: 10.1242/jcs.260685

Editor: Michael Way

Review timeline

Original submission:	4 October 2022
Editorial decision:	10 October 2022
Rebuttal Received:	10 October 2022
Editorial decision:	21 November 2022
First revision received:	6 February 2023
Accepted:	13 February 2023

Original submission

First decision letter

MS ID#: JOCES/2022/260685

MS TITLE: SARS-CoV-2 infection alkalinizes the ERGIC and lysosomes through the viroporin activity of the viral envelope protein

AUTHORS: Wen-An Want, Amado Carreras Sureda, and Nicolas Demaurex
ARTICLE TYPE: Research Article

Dear Dr. Demaurex,

Thank you for your recent submission to Journal of Cell Science. Unfortunately both an advisory board member and myself feel the manuscript would be inappropriate for JCS. Our instructions to referees indicate that we will only accept articles that provide mechanistic insight into fundamental cellular processes. While the study is well executed and the data are convincing, the SARS-CoV2 envelope protein is already well characterised. Its localization and function in increasing Golgi pH (as mentioned in the introduction) is known (Boson, B., 2021, Cabrera-Garcia, D., 2021). Given this, we feel your study does not represent sufficient advance and would be unlikely to get through our review process. In the absence of further characterization of the sorting motifs that target the protein to ERGIC we feel the paper is better suited to another journal.

JCS now accepts only about 20% of the manuscripts that it receives. We aim to let authors know quickly if we decide not to send their manuscript out for review, allowing them to resubmit to a more appropriate journal as rapidly as possible. I am sorry I cannot be more encouraging on this occasion.

© 2023. Published by The Company of Biologists under the terms of the Creative Commons Attribution License (https://creativecommons.org/licenses/by/4.0/).

Rebuttal letter

Dear Dr Way

I understand you concerns but before we submit our study to another journal I wanted to stress that our study is the first reporting organelle pH changes associated with infection with the fully replicative SARS-CoV2 virus and to link these defects to the channel function of the viral envelope protein, using pore mutants and quantitative, time-resolved measurement of the pH within the lumen of the ERGIC. Other studies used ectopic expression of viral proteins and non-selective probes.

We therefore believe that our study is suited for your journal as provides significant mechanistic insight on the host cell defects occurring during SARS-CoV-2 infection and would appreciate it if you could reconsider your decision based on these considerations.
Sincerely

Nic Demaurex

Original submission

First decision letter

MS ID#: JOCES/2022/260685

MS TITLE: SARS-CoV-2 infection alkalinizes the ERGIC and lysosomes through the viroporin activity of the viral envelope protein

AUTHORS: Wen-An Want, Amado Carreras Sureda, and Nicolas Demaurex
ARTICLE TYPE: Research Article

We have now reached a decision on the above manuscript.

To see the reviewers' reports and a copy of this decision letter, please go to: https://submit-jcs.biologists.org and click on the 'Manuscripts with Decisions' queue in the Author Area. (Corresponding author only has access to reviews.)

As you will see, both reviewers raise a number of substantial criticisms that prevent me from accepting the paper at this stage. They suggest, however, that a revised version might prove acceptable, if you can address their concerns. If you think that you can deal satisfactorily with the criticisms on revision, I would be pleased to see a revised manuscript. We would then return it to the reviewers.

Please ensure that you clearly highlight all changes made in the revised manuscript. Please avoid using 'Tracked changes' in Word files as these are lost in PDF conversion.

I should be grateful if you would also provide a point-by-point response detailing how you have dealt with the points raised by the reviewers in the 'Response to Reviewers' box. Please attend to all of the reviewers' comments. If you do not agree with any of their criticisms or suggestions please explain clearly why this is so.

Reviewer 1

Advance summary and potential significance to field

The manuscript by Wang et al analyzes the role of the SARS-CoV2 envelope protein E on the pH of the ERGIC and of lysosomes. The authors used either pH-sensitive fluorescent proteins targeted to

© 2023. Published by The Company of Biologists under the terms of the Creative Commons Attribution License (https://creativecommons.org/licenses/by/4.0/).

the ERGIC or pH-sensitive fluorescent dextrans to load lysosomes to measure the pH in situ. They conclude that the E protein provides a pathway for the passive permeation of protons that dissipate the pH gradient generated by the V-ATPase.

Comments for the author

The results are convincing though not entirely unexpected because the protein had been shown earlier to increase membrane permeability to monovalent and divalent cations and because it was reported to alkalinize lysosomes. The most novel aspect of the findings of Wang et al are the measurements of ERGIC pH and the demonstration that the viral protein induces a significant, albeit modest alkalinization. The functional significance of this pH change to viral fitness and proliferation was not established.

There are several unexpected and unexplained observations that should be addressed, as well as some technical aspects that are unclear:

1) The Sec22b-pHluorin probe accumulates in the ERGIC but is also clearly visible in what appears to be the ER. What fraction of the signal comes from the ER? Importantly, co-expression of the viral protein or infection with viral particles could alter the balance of this imperfect distribution, skewing the pH measurements towards the more alkaline pH of the ER. Indeed, viral infection or protein overexpression can cause ER stress. It is imperative for the authors to provide quantitative analysis of the colocalization of Sec22b-pHluorin with the ERGIC and ER before and after infection or E protein overexpression.

2) The lysosomal pH following viral infection is reported as 5.8 and no further change in pH was obtained when adding concanamycin. This is unexpected and rather inconsistent with most of the existing literature, where inhibition of the V-TPases raises the lysosomal pH to values approximating the cytosolic pH (i.e ≈7.0). What do the authors attribute the remaining >1 unit acidification to, if the V-ATPases have in fact been fully and properly inhibited?

3) The authors report maximal effects of pH at MOI ≤0.5. If MOI refers, as is normally the case, to multiplicity of infection (i.e. number of virions per cell), how can maximal effects be obtained with fewer than one viral particle per cell? This comment is particularly pertinent to lysosomes: each cell contains dozens, possibly hundreds of these and each viral particle could presumably only affect one of these. A maximal effect would require much larger MOIs.

4) In the control, uninfected cells concanamycin is reported to dissipate the pH by about 0.2 units only. Why is lysosomal pH not fully dissipated even under control conditions? The disagreement with the vast majority of the literature makes the measurements questionable.

5) The cells were grown in medium that contains bicarbonate. Was the same medium used for the pH measurements or was it switched to bicarbonate-free solution?

Reviewer 2

Advance summary and potential significance to field

Wang et al., present a manuscript documenting the role of SARS-CoV-2 Envelope protein in acting as a viroporin to neutralise pH at a variety of internal organelles. The strength of this manuscript is the demonstration of an alteration in organellar physiology (pH) in the context of a live infection, and the deployment of accurate pH sensors for the ERGIC. The weakness of this manuscript is that it is observational and reports findings that are not unexpected, based on prior publications (e.g., as the authors cite, Cabrera-Garcia, 2021 has already shown that expression of E could neutralise Golgi pH) and knowledge of the behaviour of Envelope from SARS-CoV as a viroporin. Elements relating to the physiological function of E (say, in ER-stress) are underdeveloped. The manuscript is brief. The luminally-placed ERGIC-targeted pHlourin is a nice tool to measure pH in this organelle, and the authors do a good job to characterise its behaviour at different pH, allowing accurate and absolute measurement of pH in these organelles – they report a slight neutralisation of ERGIC pH (c0.1 – 0.2 pH units). The tool to measure lysosomal pH is less well advanced (relying on pH-

© 2023. Published by The Company of Biologists under the terms of the Creative Commons Attribution License (https://creativecommons.org/licenses/by/4.0/).

dependent quenching of Oregon-Green dextran), but again reports a slight neutralisation of lysosomal pH (c.0.5 pH units). Although slight, the correlation of pH neutralisation in the ERGIC and lysosomes with increased MOI is clear. What the physiological relevance of these slight changes in pH is is unclear.

Comments for the author

The authors use a double mutant of E that is predicted to abrogate both a pore lining residue (Asn15Ala (not Asp15, as reported on L63)) and a residue predicted to affect oligomerisation (Val25Phe). Firstly, I don't think Val25 is within the pore domain (as cited on L144, L147) and it would make sense to switch 'oligomerisation' and 'ion conductance' on L64 to properly associate each mutation with its predicted functional effect. Could you use the data from Mandala 2020 to see where Val25 points? Secondly, as far as I am aware, I think these effects are inferred based on modelling/in-vitro data from SARS-CoV Envelope. Could the authors use their Sec22-pHlourin reporter to deconvolve the relative contribution of Asn15 and Val25 to the ion conductance of SARS-CoV-2 Envelope?

In cells co-expressing E and the pHlourin-based reporter of ERGIC-pH, a significant amount of the reporter is retained in the ER and doesn't reach the ERGIC (Figure 4B). From the overlays in Figure 4, this does at least appear to be the compartment that E localises to, but perhaps the authors could modify their conclusions to report that E is modifying the pH in this hybrid compartment rather than the ERGIC itself? If the authors want to report an effect on ERGIC pH can they retarget their probe to this organelle?

The authors observe neutralisation of lysosomal pH in infected cells, but not in cells transfected with E. This is hard to get my head around. However transfection efficiencies for VeroE6 cells are notoriously poor (the authors don't cite transfection efficiency) and to measure lysosomal pH, the authors use transient transfection of untagged versions of E in their dextran quenching assay.

It isn't clear to me how the authors can be sure that their measurement of E's (zero) effect on lysosomal pH is occurring in the transfected cells, rather than in the majority non-transfected cells. Can they clarify this? Having established the effect of live-virus on lysosomal neutralisation, I'm also not clear how necessary this data is, especially if it confuses things.

The cellular toxicity element is an important element of the manuscript, but is underdeveloped. The authors have only investigated the Val25Phe mutation in induction of the ER-stress response, not the pore mutant Asn15Ala, or the combined mutant that they used to perturb ERGIC pH. Can the authors solidify this data including all these mutants so that elements of the paper can be linked together?

Again, the authors present results from N = 2 independent experiments with technical repeats used to boost the numbers. Statistical analysis should be employed (it is absent), and the authors should investigate and provide explanation for why differential arms of the ER stress-response are activated (some in an Val25 dependent manner) when E is expressed. At what stage does E-expression lead to induction of apoptosis? The positive control for apoptosis appears to be use of a detergent (NP40 – presume this is IGEPAL?). Could the authors use a more appropriate apoptotic induction agent and assay (early/late) to determine how and when expression of E is linked to the induction of cellular toxicity (and whether this is dependent upon the ER-stress response)?

In general, I think the n-numbers for all assays in the paper should be enhanced. The majority of experiments are from 2 independent experiments, using technical repeats (4) to boost the n. There is quite a lot of noise in the technical repeats (e.g., in Fig. 2C, Fig. 3C, two populations in the 4 technical repeats appear – one population with no effect, one population with an effect. What is the true effect of the intervention? It's hard to draw conclusions from the authors' data here) and it isn't clear that statistical analysis has been applied appropriately to independent experimental means, rather than technical repeats. Can the authors boost their N-numbers, describe more fully, and deploy appropriately the statistical tests in the paper?

As a minor point, an opinion piece by Bill Gates is cited to support the claim that SARS-CoV-2 propagates more efficiently than SARS-CoV or MERS-CoV. It may just be my ignorance, but is there

© 2023. Published by The Company of Biologists under the terms of the Creative Commons Attribution License (https://creativecommons.org/licenses/by/4.0/).

evidence that CoV-2 'propagates' better than other viruses, or is this just an inference from the degree of global spread of these agents that is influenced by the NPIs as well as the inherent propagability of the virus? It would be good to back up this claim with primary data.

First revision

Author response to reviewers' comments

The reviewers' comments are in *italics*, our answers in **bold**. The page numbers refer to the revised manuscript with changes highlighted in track mode

Reviewer 1 Advance Summary and Potential Significance to Field:
The manuscript by Wang et al analyzes the role of the SARS-CoV2 envelope protein E on the pH of the ERGIC and of lysosomes. The authors used either pH-sensitive fluorescent proteins targeted to the ERGIC or pH-sensitive fluorescent dextrans to load lysosomes to measure the pH in situ. They conclude that the E protein provides a pathway for the passive permeation of protons that dissipate the pH gradient generated by the V-ATPase.

Reviewer 1 Comments for the Author: The results are convincing though not entirely unexpected because the protein had been shown earlier to increase membrane permeability to monovalent and divalent cations and because it was reported to alkalinize lysosomes. The most novel aspect of the findings of Wang et al are the measurements of ERGIC pH and the demonstration that the viral protein induces a significant, albeit modest alkalinization. The functional significance of this pH change to viral fitness and proliferation was not established.

There are several unexpected and unexplained observations that should be addressed, as well as some technical aspects that are unclear:

We thank the reviewer for the careful evaluation of our study. As suggested, we have performed additional experiments to clarify the unexpected observations and technical aspects of our work.

1)The Sec22b-pHluorin probe accumulates in the ERGIC but is also clearly visible in what appears to be the ER. What fraction of the signal comes from the ER? Importantly, co-expression of the viral protein or infection with viral particles could alter the balance of this imperfect distribution, skewing the pH measurements towards the more alkaline pH of the ER. Indeed, viral infection or protein overexpression can cause ER stress. It is imperative for the authors to provide quantitative analysis of the colocalization of Sec22b-pHluorin with the ERGIC and ER before and after infection or E protein overexpression.

Good point. We now provide a quantitative analysis of the colocalization of Sec22b-pHluorin with the ERGIC and ER markers (new Fig. 5A). The fraction of the signal colocalizing with RFP-KDEL was ~20%, while the probe colocalized more extensively with ERGIC-53 (≈60%). Importantly, the fractions of the signal colocalizing with the ER and ERGIC markers were not significantly altered by viral protein expression. This indicates that the pH changes associated with ectopic E protein expression do not reflect an increased retention of the probe in the ER. We thank the reviewer for suggesting this important control.

2)The lysosomal pH following viral infection is reported as 5.8 and no further change in pH was obtained when adding concanamycin. This is unexpected and rather inconsistent with most of the existing literature, where inhibition of the V-TPases raises the lysosomal pH to values approximating the cytosolic pH (i.e ≈7.0). What do the authors attribute the remaining >1 unit acidification to, if the V-ATPases have in fact been fully and properly inhibited?

We were also surprised by the poor pH dissipation evoked by V-ATPase inhibitors in Vero cells. To clarify this, we have compared the effect of the inhibitors in Vero and HeLa cells (new Fig. S3B). Bafilomycin evoked a rapid increase in lysosomal pH in HeLa cells but a much slower

© 2023. Published by The Company of Biologists under the terms of the Creative Commons Attribution License (https://creativecommons.org/licenses/by/4.0/).

alkalinization in Vero cells, while the protonophore CCCP caused a rapid alkalinization in both cell lines. This suggested that Vero cells have a lower proton leak than HeLa cells, prompting us to repeat the lysosomal pH measurements using longer incubation times with the inhibitors. The new Fig. S3C shows that a 30 min incubation of Vero cells with bafilomycin is required to equilibrate the lysosomal pH to pH ~7.0 as expected from the inhibition of V-ATPases. This cell-specific effect was unexpected and might underlie the ability of Vero cells to tolerate viral infection and propagation. Of note, OG-Dextran does not respond well to pH changes above pH 6 (Fig. S3A-B) and thus cannot precisely report the full pH dissipation expected to occur upon inhibition of V-ATPases.

3)The authors report maximal effects of pH at MOI ≤0.5. If MOI refers, as is normally the case, to multiplicity of infection (i.e. number of virions per cell), how can maximal effects be obtained with fewer than one viral particle per cell? This comment is particularly pertinent to lysosomes: each cell contains dozens, possibly hundreds of these and each viral particle could presumably only affect one of these. A maximal effect would require much larger MOIs.

MOI refers to the number of virions added at the start of infection per cell. With a viral cycle of 6h all cells infected at MOI 1 are expected to be infected within 24h, with each infected cell producing hundreds of copies of E protein. With these titers, enough viral particles are produced in the infected cells to anticipate a functional impact on the ionic homeostasis of the ERGIC and lysosomes.

4)In the control, uninfected cells concanamycin is reported to dissipate the pH by about 0.2 units only. Why is lysosomal pH not fully dissipated even under control conditions? The disagreement with the vast majority of the literature makes the measurements questionable.

We now show that a longer incubation with V-ATPase inhibitors is required to fully dissipate the lysosomal pH in Vero cells while a faster dissipation is observed in HeLa cells (new Figs. S3B-C). Despite this limitation, a 10 min incubation with 1 µM concanamycin increased the lysosomal pH of Vero cells by 0.6-0.8 pH units (Fig 3C). This effect is significant both in infected and uninfected cells when pH values from individual cells are statistically evaluated.

5)The cells were grown in medium that contains bicarbonate. Was the same medium used for the pH measurements or was it switched to bicarbonate-free solution?

Bicarbonate was present for pH measurements of cells infected with the virus. This was for technical reasons because the BSL3 microscope environmental chamber required continuous CO2 supply. A bicarbonate-free solution was used for pH measurements in cells expressing the viral proteins. Although the bicarbonate buffer is expected to reduce pH fluctuations, we observed that viral infection alkalinizes the ERGIC and lysosomes and believe that this enhances the physiological relevance of our findings.

Reviewer 2 Advance Summary and Potential Significance to Field:
Wang et al., present a manuscript documenting the role of SARS-CoV-2 Envelope protein in acting as a viroporin to neutralise pH at a variety of internal organelles. The strength of this manuscript is the demonstration of an alteration in organellar physiology (pH) in the context of a live infection, and the deployment of accurate pH sensors for the ERGIC. The weakness of this manuscript is that it is observational and reports findings that are not unexpected, based on prior publications (e.g., as the authors cite, Cabrera-Garcia, 2021 has already shown that expression of E could neutralise Golgi pH) and knowledge of the behaviour of Envelope from SARS-CoV as a viroporin. Elements relating to the physiological function of E (say, in ER-stress) are underdeveloped. The manuscript is brief. The luminally-placed ERGIC-targeted pHlourin is a nice tool to measure pH in this organelle, and the authors do a good job to characterise its behaviour at different pH, allowing accurate and absolute measurement of pH in these organelles – they report a slight neutralisation of ERGIC pH (c0.1 – 0.2 pH units). The tool to measure lysosomal pH is less well advanced (relying on pH-dependent quenching of Oregon-Green dextran), but again reports a slight neutralisation of lysosomal pH (c.0.5 pH units). Although slight, the correlation of pH neutralisation in the ERGIC and lysosomes with increased MOI is clear. What the physiological relevance of these slight changes in pH is is unclear.

© 2023. Published by The Company of Biologists under the terms of the Creative Commons Attribution License (https://creativecommons.org/licenses/by/4.0/).

We thank the reviewer for the thorough evaluation of our study. As suggested we have performed additional pH recording with single mutants, characterized in more detail the subcellular localization of the probe, and documented more fully the ER stress response associated with ectopic expression of the E protein. We believe that these additional data significantly enhance the physiological relevance of our study and thank the reviewer for suggesting these experiments.

Reviewer 2 Comments for the Author:
The authors use a double mutant of E that is predicted to abrogate both a pore lining residue (Asn15Ala (not Asp15, as reported on L63)) and a residue predicted to affect oligomerisation (Val25Phe). Firstly, I don't think Val25 is within the pore domain (as cited on L144, L147) and it would make sense to switch 'oligomerisation' and 'ion conductance' on L64 to properly associate each mutation with its predicted functional effect. Could you use the data from Mandala 2020 to see where Val25 points? Secondly, as far as I am aware, I think these effects are inferred based on modelling/in-vitro data from SARS-CoV Envelope. Could the authors use their Sec22-pHlourin reporter to deconvolve the relative contribution of Asn15 and Val25 to the ion conductance of SARS-CoV-2 Envelope?

As suggested, we have performed ERGIC pH recordings in Vero cells expressing the single and double mutants to establish the relative contribution of Asn15 and Val25 to the ion conductance of the envelope protein. These new data show that the ERGIC pH is impacted by the substitution of Val25, but not of Asn15 (Fig. 5B). This suggests that the Val25 residue is more critical for proton permeation. We thank the reviewer for suggesting this experiment.

We mutated N15 and V25 because both residues are pore-facing in the single pentamer model of Mandala et al. derived from NMR structures of the SARS-COV-2 envelope protein reconstituted in lipid bilayers ((Mandala et al., 2020), Fig. 3 and Extended Data Fig. 7b-e, Supplementary Table 4). In a subsequent NMR study, the same authors showed that Val25 forms an interhelical contact with Phe20 in conditions that promote channel opening (Medeiros-Silva et al., 2022). They proposed that Val25 mutations disrupt an aromatic gating ring, shifting the channel to the closed state. Other studies report an interhelical orientation for the side chain of V25 (Surya et al., 2018) with aromatic residue F26 pointing inward to constrict the pore (Mehregan et al., 2022). We have modified the text on lines 63-64 as suggested and mention these structural studies in the introduction and discussion sections (lines 73-75 and 217-225).

In cells co-expressing E and the pHlourin-based reporter of ERGIC-pH, a significant amount of the reporter is retained in the ER and doesn't reach the ERGIC (Figure 4B). From the overlays in Figure 4, this does at least appear to be the compartment that E localises to, but perhaps the authors could modify their conclusions to report that E is modifying the pH in this hybrid compartment, rather than the ERGIC itself? If the authors want to report an effect on ERGIC pH, can they retarget their probe to this organelle?

A similar point was raised by Reviewer #1. To clarify the identity of the compartment measured by the pH probe, we now provide a quantitative analysis of the colocalization of Sec22b-pHluorin with ERGIC and ER markers. As shown in the new Fig. 5AB, the fraction of the signal originating from ER structures was small (~20%) and was not significantly altered by viral protein expression. The pH probe co-localized much better with the ERGIC-53 immunoreactivity (~60%). We have also quantified the impact of E protein levels on the extent of its co-localization with ERGIC-53. This revealed that E protein remains localized in the ERGIC-53 compartment regardless of its expression level (new Fig. 4C). We had attempted to create a "more ERGIC" probe using ERGIC-53 as a targeting sequence, but the expression pattern of this construct was too variable, and we feel that the design and validation of yet another pH sensor is beyond the scope of this study.

The authors observe neutralisation of lysosomal pH in infected cells, but not in cells transfected with E. This is hard to get my head around. However, transfection efficiencies for VeroE6 cells are notoriously poor (the authors don't cite transfection efficiency) and to measure lysosomal pH, the authors use transient transfection of untagged versions of E in their dextran quenching assay.
It isn't clear to me how the authors can be sure that their measurement of E's (zero) effect on lysosomal pH is occurring in the transfected cells, rather than in the majority non-transfected

© 2023. Published by The Company of Biologists under the terms of the Creative Commons Attribution License (https://creativecommons.org/licenses/by/4.0/).

cells. Can they clarify this? Having established the effect of live-virus on lysosomal neutralisation, I'm also not clear how necessary this data is, especially if it confuses things.

The proportion of Vero cells immunoreactive for the transfected E-Strep epitope averaged 26%, measured by flow cytometry. In these conditions, a lysosomal alkalinization should be apparent in the cell population or at least in a fraction of transfected cells. Our observation that the lysosomal pH is unaffected indicates that ectopic E protein expression does not recapitulate the effect of viral infection on the ionic homeostasis of lysosomes. The simplest explanation is that overexpressed E does not reach lysosomes, as suggested by its extensive colocalization with the ERGIC marker. Viral infection might reprogram the cellular secretory pathway to target the viral particles to lysosomes for egress. We believe that these data provide interesting mechanistic insight on the trafficking of the envelope protein and would like to keep them in the manuscript.

The cellular toxicity element is an important element of the manuscript, but is underdeveloped. The authors have only investigated the Val25Phe mutation in induction of the ER-stress response, not the pore mutant Asn15Ala, or the combined mutant that they used to perturb ERGIC pH. Can the authors solidify this data, including all these mutants so that elements of the paper can be linked together? Again, the authors present results from N = 2 independent experiments with technical repeats used to boost the numbers. Statistical analysis should be employed (it is absent), and the authors should investigate and provide explanation for why differential arms of the ER stress-response are activated (some in an Val25 dependent manner) when E is expressed. At what stage does E-expression lead to induction of apoptosis? The positive control for apoptosis appears to be use of a detergent (NP40 - presume this is IGEPAL?). Could the authors use a more appropriate apoptotic induction agent and assay (early/late) to determine how and when expression of E is linked to the induction of cellular toxicity (and whether this is dependent upon the ER-stress response)?

The cell death and ER stress assays were performed to exclude an acute toxicity associated with the ectopic expression of the E protein. Short-term (24h) expression of E did not induce apoptosis or ER stress in HEK-293 and Vero E6 cells, ruling out acute cellular toxicity in the conditions used for the pH measurements. As suggested, we have documented the ER stress response induced by long-term (72h) expression of the E protein in Vero E6 cells, using standard luciferase assays and tunicamycin as control. We co-expressed wild-type E with reporters for IRE1/ATF6 (UPRE luciferase), GRP78 (ERSE luciferase) and PERK activity (AARE-luciferase). Contrary to our earlier observations in HEK-293 cells, prolonged E protein expression did not cause ER stress in Vero E6 cells. Instead, it slightly reduced basal UPRE activity, the other two arms of the ER stress response remaining unaffected. Moreover, enforced expression of E for 72h did not alter the ER stress response induced by tunicamycin (new Fig. S4B). These data indicate that E protein expression does not sensitize Vero E6 cells to ER stress. Studying the impact of the mutants in these conditions is not justified and we did not investigate this pathway further.

We have also measured the effects of long-term (72h) expression of E on cell death, using Annexin V positivity and 7-AAD staining to quantify the proportion of apoptotic and necrotic cells. Long-term expression of WT-E potentiated apoptosis but not necrosis, mimicking the effects of thapsigargin. Unexpectedly, this effect was also observed with the V25F mutant but not with the N15A and N15A/V25F mutants (Rebuttal Figure 1). This observation cannot be easily related to the pH dissipating effects of short-term expression of the viroporin. Establishing the underlying mechanism of the delayed effects of E expression on cell death will require considerable efforts. We would therefore like to omit these data and present them in a subsequent study as they detract from the main focus of our paper on ERGIC pH.

NOTE: We have removed unpublished data that had been provided for the referees in confidence.

Rebuttal Figure 1: Effect of long-term E protein expression on apoptosis and necrosis. *Proportion of Vero E6 cells in early apoptosis (Annexin V only), late apoptosis (Annexin V and 7-AAD) and necrosis (7-AAD only) 72h after transfection with E-WT or empty vector. Cells were treated or not for the last 24h with 100nM Thapsigargin. Anova Dunnett's multiple comparisons*

© 2023. Published by The Company of Biologists under the terms of the Creative Commons Attribution License (https://creativecommons.org/licenses/by/4.0/).

test, N=3. Bottom, Annexin V positivity in Vero E6 cells transfected with the indicated constructs. Anova Dunnett's multiple comparisons test. N=3

In general, I think the n-numbers for all assays in the paper should be enhanced.
The majority of experiments are from 2 independent experiments, using technical repeats (4) to boost the n. There is quite a lot of noise in the technical repeats (e.g., in Fig. 2C, Fig. 3C, two populations in the 4 technical repeats appear – one population with no effect, one population with an effect. What is the true effect of the intervention? It's hard to draw conclusions from the authors' data here) and it isn't clear that statistical analysis has been applied appropriately to independent experimental means, rather than technical repeats. Can the authors boost their N-numbers, describe more fully, and deploy appropriately the statistical tests in the paper?

The pH data from infected cells reported the averaged value of multiple cells (n=43-240) imaged in individual wells (n=4-6) of different plates (n=2-3) on the BSL3 microscope. Wells were considered as independent samples to enable statistical analysis. This presentation mode reduces n and generates noise as a variable number of cells is sampled in each well. To fully exploit the information recorded, we now report the pH values of all the individual cells imaged and perform the statistical analysis on the pooled cell populations. The number of cells imaged is now indicated in the Figure legends

As a minor point, an opinion piece by Bill Gates is cited to support the claim that SARS-CoV-2 propagates more efficiently than SARS-CoV or MERS-CoV. It may just be my ignorance, but is there evidence that CoV-2 'propagates' better than other viruses, or is this just an inference from the degree of global spread of these agents that is influenced by the NPIs as well as the inherent propagability of the virus? It would be good to back up this claim with primary data.

The cited opinion piece written at the onset of the pandemic referred to the more efficient transmission of the disease among human populations, not to the inherent propagability of the virus. We have now replaced this reference by a more recent review comparing the characteristics and attributes of the coronaviruses SARS-CoV-1, MERS-CoV, and SARS-CoV-2 (Goyal et al., 2022).

References cited:

Goyal, R., Gautam, R.K., Chopra, H., Dubey, A.K., Singla, R.K., Rayan, R.A., and Kamal, M.A. (2022). Comparative highlights on MERS-CoV, SARS-CoV-1, SARS-CoV-2, and NEO-CoV. EXCLI J *21*, 1245-1272.
Mandala, V.S., McKay, M.J., Shcherbakov, A.A., Dregni, A.J., Kolocouris, A., and Hong, M. (2020). Structure and drug binding of the SARS-CoV-2 envelope protein transmembrane domain in lipid bilayers. Nat Struct Mol Biol *27*, 1202-1208.
Medeiros-Silva, J., Somberg, N.H., Wang, H.K., McKay, M.J., Mandala, V.S., Dregni, A.J., and Hong, M. (2022). pH- and Calcium-Dependent Aromatic Network in the SARS-CoV-2 Envelope Protein. J Am Chem Soc *144*, 6839-6850.
Mehregan, A., Perez-Conesa, S., Zhuang, Y., Elbahnsi, A., Pasini, D., Lindahl, E., Howard, R.J., Ulens, C., and Delemotte, L. (2022). Probing effects of the SARS-CoV-2 E protein on membrane curvature and intracellular calcium. Biochim Biophys Acta Biomembr *1864*, 183994.
Surya, W., Li, Y., and Torres, J. (2018). Structural model of the SARS coronavirus E channel in LMPG micelles. Biochim Biophys Acta Biomembr *1860*, 1309-1317.

<u>Second decision letter</u>

MS ID#: JOCES/2022/260685

MS TITLE: SARS-CoV-2 infection alkalinizes the ERGIC and lysosomes through the viroporin activity of the viral envelope protein

AUTHORS: Wen-An Want, Amado Carreras Sureda, and Nicolas Demaurex

© 2023. Published by The Company of Biologists under the terms of the Creative Commons Attribution License (https://creativecommons.org/licenses/by/4.0/).

ARTICLE TYPE: Research Article

We have heard back from the original two reviews and I am happy to tell you that your manuscript has been accepted for publication in Journal of Cell Science, pending standard ethics checks.

Reviewer 1

Advance summary and potential significance to field

The authors have adequately addressed my original concerns. As far as I am concerned, the revised manuscript is now acceptable for publication in its present form.

Comments for the author

The authors have adequately addressed my original concerns. As far as I am concerned, the revised manuscript is now acceptable for publication in its present form.

Reviewer 2

Advance summary and potential significance to field

Shows that ERGIC and lysosomes are deacidified in context of SARS-CoV-2 infection and suggests E is the viroporin.

Comments for the author

The authors have made changes to the manuscript and removed some of the harder to understand data. Happy to recommend acceptance.

© 2023. Published by The Company of Biologists under the terms of the Creative Commons Attribution License (https://creativecommons.org/licenses/by/4.0/).
